# Role of proinflammatory cytokines on expression of vitamin D metabolism and target genes in colon cancer cells^☆^

**DOI:** 10.1016/j.jsbmb.2013.09.017

**Published:** 2014-10

**Authors:** Doris M. Hummel, Irfete S. Fetahu, Charlotte Gröschel, Teresa Manhardt, Enikő Kállay

**Affiliations:** Department of Pathophysiology and Allergy Research, Medical University of Vienna, Währinger Gürtel 18-20, 1090 Vienna, Austria

**Keywords:** IBD, inflammatory bowel disease, CYP24A1, 1,25-dihydroxyvitamin D_3_ 24-hydroxylase, CYP27B1, 25-hydroxyvitamin D_3_ 1α-hydroxylase, CYP3A4, cytochrome P450, family 3, subfamily A, polypeptide 4, TRPV6, transient receptor potential cation channel, subfamily V, member 6, IGFBP3, insulin-like growth factor binding protein 3, PGE2, prostaglandin E2, COX-2, cyclooxygenase-2, 15-PGDH, 15-hydroxyprostaglandin dehydrogenase, Vitamin D, Inflammation, TNFα, IL-6, CYP24A1, TRPV6

## Abstract

Interleukin 6 (IL-6) and tumor necrosis factor alpha (TNFα) are proinflammatory cytokines that play a critical role in inflammatory bowel disease, as well as in colorectal tumorigenesis. We hypothesize that these cytokines modulate the expression and thus activity of the vitamin D system in colonic epithelial cells. We treated the colon cancer cell line COGA-1A for 6, 12, and 24 h with 1,25-dihydroxyvitamin D_3_ (1,25-D_3_), IL-6, TNFα, and with combinations of these compounds. Using quantitative RT-PCR, we analyzed mRNA expression of genes activating and catabolizing 1,25-D_3_ (1α-hydroxylase (CYP27B1), 24-hydroxylase (CYP24A1)), expression of several vitamin D target genes, as well as expression of cyclooxygenase 2 (COX-2) and 15-hydroxyprostaglandin dehydrogenase. As expected, treatment with 1,25-D_3_ resulted in an upregulation of CYP24A1, whereas expression of CYP27B1 was not affected. Treatment with TNFα and IL-6 led to decreased expression of the vitamin D activating enzyme CYP27B1. The strong inflammatory property of TNFα was mirrored by its activation of COX-2 and inhibition of prostaglandin E2 (PGE2) catabolism. Interestingly, expression of the calcium ion channel TRPV6 was markedly decreased by TNFα.

We conclude from these results that the presence of proinflammatory cytokines might impair activation of 1,25-D_3_, limiting its anti-inflammatory action.

This article is part of a Special Issue entitled ‘16th Vitamin D Workshop’.

## Highlights

• TNFα decreases CYP27B1 mRNA expression.
• TNFα inhibits transcription of the calcium ion channel TRPV6.
• 1,25-D_3_ inhibits TNFα-induced upregulation of COX-2.

## 1 Introduction

The term inflammatory bowel disease (IBD) describes a set of diseases characterized by chronic inflammation of the intestinal mucosa. The two major forms of IBD are ulcerative colitis and Crohn's disease [1]. The highest incidence rates of IBD have been reported in the UK, northern Europe, and North America [2]. This geographical distribution points toward an influence of sunlight exposure on the prevalence of this disease.

The proinflammatory cytokines TNFα and IL-6 play a crucial role in IBD [3], [4] and treatment with TNFα-blockers is a standard therapy for ulcerative colitis. Patients suffering from IBD have a higher risk to develop colorectal cancer (CRC). In CRC, both TNFα and IL-6 are often overexpressed [5], [6].

Increasing evidence supports the preventive effect of vitamin D on the development of IBD and CRC [7], but how inflammation affects the local vitamin D system in the colon is less known. In the present study, on the one hand, we examined the influence of two proinflammatory cytokines on the expression of genes involved in vitamin D metabolism, such as CYP27B1, the vitamin D activating enzyme [8], and CYP24A1, the vitamin D catabolizing enzyme. On the other hand, we assessed whether treatment with TNFα and IL-6 would impair the effect of 1,25-dihydroxyvitamin D_3_ (1,25-D_3_) on different vitamin D target genes. We have chosen CYP3A4 (one of the crucial drug-metabolizing enzymes), the calcium channel TRPV6, and the insulin-like growth factor binding protein, IGFBP3, all well-known targets of 1,25-D_3_ [9], [10], [11].

## 2 Materials and methods

### 2.1 Cell culture

The adenocarcinoma cell line COGA-1A is derived from a moderately differentiated pT3 colon tumor and was characterized previously [6], [12]. One week after confluency, COGA-1A cells were treated with 10 nM 1,25-D_3_, 100 ng/ml IL-6, 50 ng/ml TNFα, or with combinations of these compounds for 6, 12, and 24 hours (h). Controls were treated with PBS and 0.01% EtOH.

### 2.2 RNA extraction and reverse transcription (RT)

Total RNA was isolated using TRIzol reagent (Invitrogen, Grand Island, NY, USA) according to the manufacturer's instructions. Integrity of the RNA was analyzed on agarose gels by staining with GelRed (Biotium, Hayward, CA, USA). 2 μg of total RNA was reverse transcribed using RevertAid H Minus Reverse Transcriptase and Random Hexamer Primers following the manufacturer's protocol (Fermentas, Ontario, Canada).

### 2.3 Quantitative real time RT-PCR

Quantitative real time RT-PCR (qRT-PCR) was performed as described before [13]. We normalized expression of the target genes to the expression of the housekeeping gene Beta-2-Microglobulin (B2M) and set relative to the calibrator (total human RNA, Clontech, Mountain View, CA, USA) to calculate relative expression with the ΔΔCt method. Sequences for B2M [14], CYP24A1 [15], CYP27B1 [15], and cytochrome P450 3A4 (CYP3A4) [16] have been described previously. Primer sequences for insulin-like growth factor binding protein (IGFBP3) were: forward: CAGAATATGGTCCCTGCCG; reverse: GGGACTCAGCACATTGAGG; COX-2: forward: GCCCTTCCTCCTGTGCCT; reverse: CAGGAAGCTGCTTTTTACCTTTG; 15-PGDH: forward: TGCTTCAAAGCATGGCATAG; reverse: AACAAAGCCTGGACAAATGG. Transient receptor potential cation channel, subfamily V, member 6 (TRPV6) mRNA expression was determined using TaqMan Gene Expression Assay (Cat. # 4331182, Life Technologies, Carlsbad, CA, USA).

### 2.4 Statistical analysis

We used SPSS statistics package, version 18.0 for statistical analysis and GraphPadPrism 5.0 for drawing the figures. We performed one-way ANOVA on log-transformed data with Tukey's post hoc test for multiple comparisons.

## 3 Results

### 3.1 Regulation of CYP24A1 and CYP27B1 expression by 1,25-D_3_, TNFα, and IL-6

**Fig. 1:**
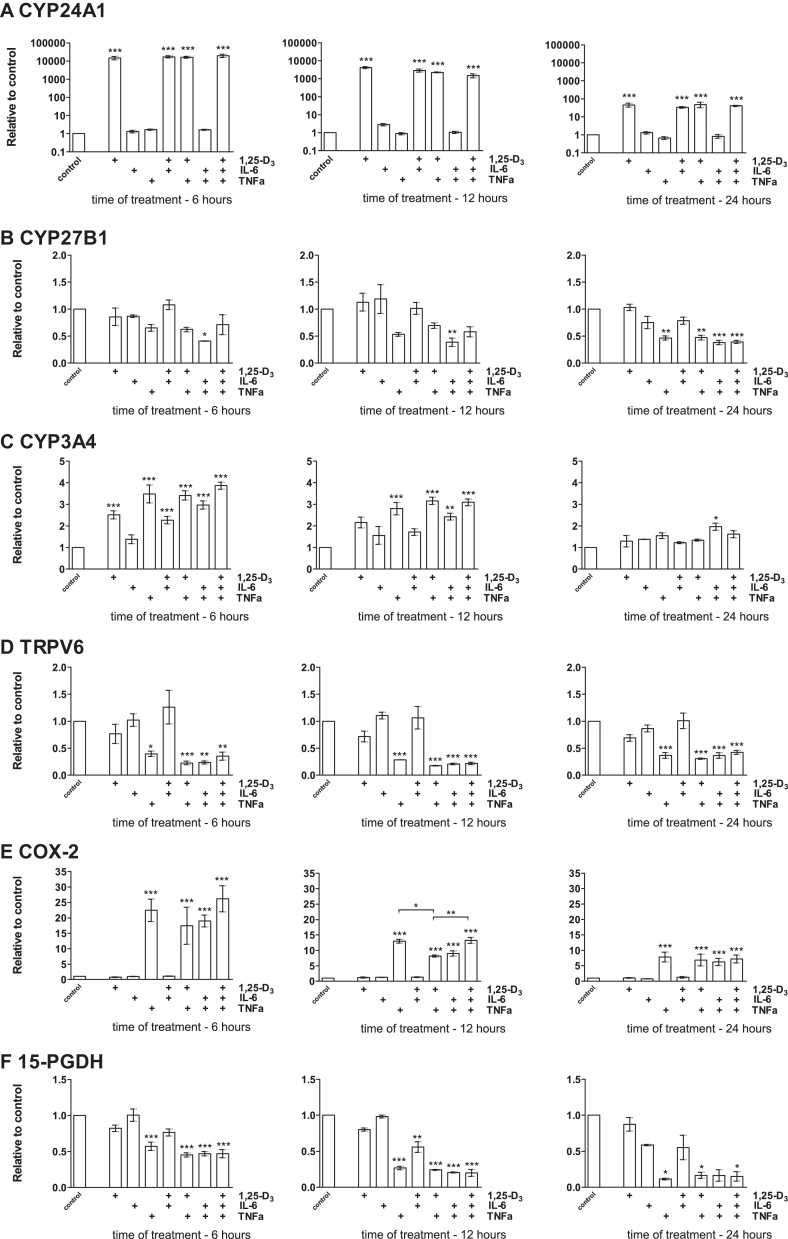
Impact of 1,25-D_3_, IL-6, and TNFα on CYP24A1, CYP27B1, CYP3A4, TRPV6, COX-2, and 15-PGDH expression. Cells were treated with 1,25-D_3_, IL-6, TNFα, and combinations of these compounds for 6 h, 12 h, and 24 h. mRNA expression of CYP24A1 (A), CYP27B1 (B), CYP3A4 (C), TRPV6 (D), COX-2 (E), and 15-PGDH (F) was assessed by qRT-PCR. Each experiment was set relative to the vehicle control. Columns represent mean of 3 independent experiments, bars indicate SEM. Asterisks indicate statistical significant difference compared with vehicle control (*p < 0.05, **p < 0.01, ***p < 0.001).

As expected, treatment of COGA-1A cells with 1,25-D_3_ led to a marked increase in the expression of the vitamin D degrading enzyme CYP24A1 (15.000-fold increase after 6 h compared with control) but the expression of the vitamin D activating enzyme CYP27B1 remained constant (Fig. 1A and B). IL-6 treatment for 12 h increased CYP24A1 expression almost three times. TNFα upregulated CYP24A1 expression 1.7-fold after 6 h, however, the increase did not reach statistical significance (Fig. 1A).

TNFα reduced mRNA expression of the 1,25-D_3_ synthesizing enzyme CYP27B1, both alone and in combination, at all time-points. After 24 h the effect of TNFα alone became highly significant, reducing CYP27B1 levels to 46% of the vehicle control. At earlier time-points, only the combination with IL-6 was highly effective and reduced CYP27B1 expression by 60% after 6 h and 12 h (Fig. 1B).

### 3.2 Effect of 1,25-D_3_, IL-6, and TNFα on expression of the vitamin D target genes CYP3A4, TRPV6, IGFPB3, COX-2, and 15-PGDH

1,25-D_3_ increased CYP3A4 mRNA expression after 6 h 2.5-fold, but the expression of TRPV6 remained unchanged. IL-6 treatment has not affected CYP3A4 or TRPV6 expression at any time-point. Surprisingly, treatment with TNFα had a strong effect on expression of the vitamin D target genes CYP3A4 and TRPV6. It increased CYP3A4 expression even more than 1,25-D_3_ treatment already after 6 h, irrespective whether it was applied alone (3.5-fold increase) or in combinations. After 12 h, the increase was only 2.8-fold, returning to normal after 24 h (Fig. 1C). In contrast, expression of TRPV6 was strongly inhibited by TNFα in all combinations at all time-points with a maximal reduction after 12 h (28% of the vehicle-treated control, Fig. 1D). Neither 1,25-D_3_, nor IL-6 or TNFα affected IGFPB3 expression in these cells (data not shown).

COX-2 and 15-PGDH expression was unresponsive to 1,25-D_3_ treatment in COGA-1A cells. IL-6 reduced 15-PGDH mRNA expression after 24 h by 41%, but had no influence on COX-2 expression. As expected, TNFα highly increased COX-2 expression (22-fold after 6 h) and decreased 15-PGDH mRNA levels (9-fold after 24 h) at all investigated time-points both alone and in all treatment combinations. 1,25-D_3_ was able to reduce TNFα-induced COX-2 expression after 12 h by 37%. This inhibitory effect was lost when IL-6 was also added to the TNFα and 1,25-D_3_ treatment. Interestingly, the combination of 1,25-D_3_ and IL-6 led to a 44% downregulation of 15-PGDH mRNA level after 12 h, whereas COX-2 expression remained stable (Fig. 1E and F).

## 4 Discussion

The anti-inflammatory effects of 1,25-D_3_ on IBD have been studied extensively [7], [17], however, whether activation and degradation of vitamin D is impaired by an existing inflammation is not yet clear. In this study, we show for the first time that TNFα significantly reduced CYP27B1 mRNA expression and expression of the calcium ion channel TRPV6 in colorectal cancer cells. Whether this reduces the capacity of the cells to activate vitamin D needs to be proven.

The vitamin D degrading enzyme CYP24A1 is one of the main target genes of 1,25-D_3_. Overexpression of this enzyme likely leads to insensitivity of the tissue toward 1,25-D_3_, limiting its anti-proliferative and pro-apoptotic functions [18]. We have shown previously, that both CYP24A1 expression and activity in COGA-1 cells is highly inducible by 1,25-D_3_ [19]. In our experiments, CYP24A1 was massively induced already after 6 h of treatment with 1,25-D_3_. This induction decreased with time but remained more than 35-fold higher even after 24 h treatment. As CYP24A1 expression is paralleled with high enzymatic activity, this would explain the lack of an 1,25-D_3_ effect on the other known VDR target genes such as TRPV6 and IGFBP3. We also observed a slight increase in CYP24A1 expression after treatment with the inflammatory cytokines. Whether such a 2–3-fold increase in CYP24A1 mRNA expression has any physiological meaning, remains questionable.

Several studies reported downregulation of CYP27B1 promoter activity and mRNA expression upon treatment with 1,25-D_3_, however, data are inconsistent and seem to depend on tissue type and state of cell differentiation [20], [21]. In the COGA-1A cells, CYP27B1 mRNA expression remained constant after 1,25-D_3_ treatment, TNFα, however, reduced CYP27B1 expression significantly. The human CYP27B1 promoter has numerous NFκB-binding sites [22]. Ebert et al. have shown that upon NFκB-binding, the activity of the CYP27B1 promoter strongly decreased [23]. We are the first to show in a colon cancer cell line, that TNFα inhibits CYP27B1 transcription. Whether this is mediated by NFκB needs to be proven. Combining TNFα with IL-6 repressed further CYP27B1 expression, suggesting an interplay of these two cytokines in regulation of the vitamin D system. In patients with Crohn's disease as well as in a mouse model of chemically induced inflammatory bowel disease, CYP27B1 expression was enhanced in granulomatous or lymphoid tissue. It is likely that it serves as a defense mechanism, since CYP27B1 knockout animals have more severe symptoms [24], [25].

To evaluate whether increased CYP24A1 expression leads to diminished VDR signaling, we analyzed mRNA expression of several known 1,25-D_3_ target genes, namely CYP3A4 [9], TRPV6 [10], and IGFBP3 [11].

CYP3A4 is one of the most important drug-metabolizing enzymes in humans, and its expression can be induced by 1,25-D_3_; this enzyme is also able to degrade 1,25-D_3_ [26]. In our experiments, 1,25-D_3_ treatment increased CYP3A4 levels, however, this effect was lost after 24 h. Interestingly, after 6 h, CYP3A4 expression was stronger enhanced by TNFα than by 1,25-D_3_. This rapid induction suggests a direct, probably NFκB-dependent induction of CYP3A4 transcription. In several previous studies CYP3A4 is rather inhibited by TNFα. In primary human hepatocytes TNFα-dependent NFκB activation released the PXR–RXRα-complex from the CYP3A4 promoter, suppressing CYP3A4 transcription [27]. In our study, all treatments in which 1,25-D_3_ or TNFα were present led to an upregulation of CYP3A4 after 6 h. We hypothesize that the upregulation of CYP3A4 by TNFα in COGA-1A cells might be mediated by direct binding of activated NFκB to its two putative binding sites located 2000 basepairs upstream of the start codon [28], [29].

TRPV6 is a calcium ion channel essential for the absorption of calcium from the intestinal lumen regulated by 1,25-D_3_ treatment in most CRC cells [30]. Surprisingly, in our cell line, treatment with 1,25-D_3_ had no effect on TRPV6 expression. Huybers et al. observed that in TNF^ΔARE/+^ mice, which are characterized by enhanced TNFα serum levels, TRPV6, calbindin D9k, and PMCA1b were downregulated [31]. Similarly, in our cells TRPV6 levels were affected only by TNFα. Our data suggest that inflammatory cytokines might impair calcium uptake by reducing TRPV6 levels during intestinal inflammation.

1,25-D_3_ plays a pivotal role in regulation of the prostaglandin pathway, affecting both PGE2 synthesis by inhibition of COX-2 expression, as well as its degradation by stimulation of 15-PGDH expression [32], [33]. In our cells, 1,25-D_3_ inhibited TNFα-induced upregulation of COX-2 after 12 h. Besides this effect, treatment with 1,25-D_3_ did not alter expression of COX-2 or of 15-PGDH, suggesting that the influence of 1,25-D_3_ on the PGE2-pathway is time- and tissue-dependent.

We conclude that inflammation interferes with the vitamin D metabolism. We could show that the proinflammatory cytokines TNFα and IL-6 inhibited the expression of the vitamin D activating gene CYP27B1 in the COGA-1A cell line. The inhibitory effect of TNFα on CYP27B1 and TRPV6 expression in colon cancer cells might alter calcium uptake in the inflamed intestine.
